# Enhancing Perceptual—Motor Skills in Sports: The Role of Ecological Sounds

**DOI:** 10.3390/jintelligence12020015

**Published:** 2024-01-30

**Authors:** Tiziano Agostini, Fabrizio Sors, Mauro Murgia, Alessandra Galmonte

**Affiliations:** 1 Department of Life Sciences, University of Trieste, 34127 Trieste, Italy; fsors@units.it (F.S.); mauromurgia@units.it (M.M.); 2 Department of Medicine, Surgery and Health Sciences, University of Trieste, 34127 Trieste, Italy; agalmonte@units.it

**Keywords:** auditory information, sonification, ecological sound, sport, rehabilitation, Gestalt

## Abstract

Starting approximately from the beginning of the new millennium, a series of studies highlighted that auditory information deriving from biological motion can significantly influence the behavioral, cognitive and neurophysiological processes involved in the perception and execution of complex movements. In particular, it was observed that an appropriate use of sounds deriving from one’s own movement promotes improvements in the movement execution itself. Two main approaches can be used, namely the sonification one or the ecological sound one; the former is based on the conversion of physiological and/or physical movement data into sound, while the latter is based on the use of auditory recordings of movement sounds as models. In the present article, some of the main applications of both approaches—especially the latter—to the domains of sport and motor rehabilitation are reviewed, with the aim of addressing two questions: Is it possible to consider rhythm as a Gestalt of human movement? If so, is it possible to build up cognitive strategies to improve/standardize movement performance from this Gestalt? As with most topics in science, a definitive answer is not possible, yet the evidence leads us to lean toward a positive answer to both questions.

## 1. Introduction

The proper functioning of motor systems from moment to moment depends on the continuous availability of sensory information from the visual, auditory, proprioceptive and vestibular systems, which provide information about objects in the environment and about the spatial relationships between our body and objects; this information is fundamental to the planning and refinement of movements during performance. Numerous experimental studies on various forms of locomotion in both vertebrates and invertebrates have demonstrated that all organisms share the same basic organizational principles, namely the existence of intrinsic nervous networks that are able to produce oscillatory activities and that are activated and modulated by both afferent signals and signals from superior motor centers; afferent information is used to counteract disturbances brought on by outside interference (Pearson and Gordon 2013).

Bernstein, between the years 1930 and 1940, linked psychology and physiology by combining movement behavior observation with neurophysiologic and neuromuscular features (Nicoletti 1992). For some decades thereafter, psychologists paid attention only sporadically to the integration of perception and action, with an increasing interest toward this topic around the last decades of the 20th century (e.g., Kelso and Kay 1987; Milner and Goodale 1995), when a large amount of research was conducted (Guastello 2006; Rosenbaum 2005, 2006). In the last thirty years, the study of human movement and motor control has increasingly taken on an independent and multidisciplinary identity, just as it did for neurosciences in the past, thanks to the developing connections between psychology and neurophysiology.

The relationships that exist between perception and action are crucial to understanding and controlling motor activity, so both perception and action should be considered a single functional system (Arbib 1987; Kelso et al. 1990; Kelso and Kay 1987; Lee and Young 1986; Warren 1988). If movement is, on the one hand, a way to adapt the external world to an internal goal, on the other hand, the environment can have a significant impact on how the motor act is performed by requiring an essential adaptation in relation to external parameters; as a result, accurate perception of the external world is required for the appropriate performance of any movement.

In the literature investigating the behavioral, cognitive and neurophysiological processes involved in the perception and execution of complex movements, the visual domain has received more attention than the auditory domain. However, starting approximately from the beginning of the new millennium, there has been a growing interest in the role of auditory information deriving from biological motion, as a series of studies highlighted that such a source of information can influence the abovementioned processes to a significant extent. In particular, it was observed that the use of rhythmic sounds deriving from one’s own movement can be used as a means to facilitate motor learning, to reduce the perceived exertion and to promote performance standardization/improvement (for a review, see Schaffert et al. 2019).

These studies stem from the established finding that the auditory modality is more effective than the visual modality in processing temporal—as opposed to spatial—information. This is well known for simple rhythmic movements (e.g., Repp and Penel 2002), and it is also becoming evident for the complex movements characterizing sport practice. In this regard, Murgia et al. (2017) observed that expert performers perceive temporal deviations from correct sequences more accurately through the auditory modality than through the visual modality. In relation to this, it was also observed that expert athletes are able to recognize the sound deriving from their own movement among sounds deriving from the same movement performed by other athletes (Kennel et al. 2014; Murgia et al. 2012), as well as to perceive opponents’ movement intentions through sound alone (Camponogara et al. 2017). Further evidence is provided by neurophysiological studies. For example, different patterns of brain activation are recorded for athletes listening to self-produced sports sounds than to sounds produced by other athletes (Justen et al. 2014). Moreover, sports sounds promote the activation of brain areas involved in the execution of complex movements based on expertise (Woods et al. 2014), and activation is also modulated by other factors, such as the intentionality or incidentality of the sounds, as well as their (a)synchronization with corresponding visual stimuli (Heins et al. 2020; Schmitz et al. 2013).

Sounds deriving from one’s own movement seem to convey “whole” information; the appropriate use of movement information through sounds may be helpful to athletes in terms of motor learning and performance enhancement. These benefits can be promoted using different approaches. For instance, previous studies investigated the efficacy of movement sonification (e.g., Effenberg 2005), while others focused on the use of ecological sounds (e.g., Agostini et al. 2004). In the present article, we briefly discuss the former and then focus in more detail on the latter.

In the paper titled “Rhythm, a Gestalt of human movement?” (Righi et al. 2006), published in *Gestalt Theory* 18 years ago, the role of auditory perception in sport was addressed. In that article, two questions were raised: Is it possible to consider rhythm as a Gestalt of human movement? If so, is it possible to build up cognitive strategies to improve/standardize movement performance from this Gestalt? Since then, as mentioned above, several studies investigated the role of sound in sport, and the knowledge on this topic has significantly increased. In the present article, we aim to address these “old” questions by reviewing the literature also in light of studies published more recently. These questions are in line with one of the points of the Special Issue “Grounding Cognition in Perceptual Experience”, namely the role of perceptual information in joint action coordination, action planning and action recognition in ecological situations.

## 2. The Sonification Approach

One of the approaches used to enhance sport performance through sound is the sonification of movement, namely the transformation of movement data into audio signals. More specifically, sonification has been defined as “the mapping of physiological and physical data onto psychoacoustic parameters (i.e., loudness, pitch, timbre, harmony and rhythm) in order to provide online and/or offline access to biomechanical information otherwise not available” (Schaffert et al. 2019, p. 4). Using sonification, it is possible to provide athletes with feedback on their own movement, including when performing movements that naturally do not produce any audible sound (Effenberg 2005). This type of feedback aims to increase self-awareness of movement execution, facilitating the regulation and control of movements themselves.

Typically, movement parameters of athletes or their instruments are converted into sounds to provide augmented feedback to athletes. In the literature, several examples of sonification in different sports are reported; for instance, the hydrodynamic pressure at hand paddles in swimming (Chollet et al. 1988), the acceleration of the boat in rowing (Schaffert and Mattes 2016) and the crank moment in cycling (Sigrist et al. 2016) have been converted into sounds and provided online to athletes. In the majority of studies, the relevant dimensions are mapped to provide an actual representation of one’s own performance, while in other studies, movement errors are sonified to highlight a deviation from a standard (for some examples, see Sigrist et al. 2013). The most employed auditory dimension for sonification is pitch, although several other dimensions have been reported in the literature (for a review, see Dubus and Bresin 2013). Taking advantage of the phenomenological similarity between auditory and movement structures, various correspondences can be exploited; for instance, the relative duration of sounds may correspond to the relative duration of a phase of movement. 

As for its applications, the use of augmented feedback through sonification has been tested in different sports, and it seems particularly effective in sports characterized by cyclic movements, such as rowing, cycling and swimming (although other sport applications have also been reported in the literature). For instance, Schaffert and Mattes (2016) investigated the effect of real-time sonification feedback on elite rowers. The acceleration of the boats was converted into tone pitch (i.e., the acceleration of the boat produced an increasing tone pitch), and the sound was provided to the rowers. Examining the performances of athletes in different conditions with and without auditory feedback, they found significant improvements due to auditory feedback in several performance parameters (e.g., speed), with no concurrent higher effort exerted by participants. Similar results were previously obtained by Schaffert et al. (2011), suggesting the replicability of this evidence.

The use of sonification to improve complex movements is not limited to sports; indeed, it has been reported in motor rehabilitation as well. In this field, since the nineties, the use of audio-based techniques such as rhythmic auditory stimulation (RAS) has proven effective in different categories of patients with neurological disorders (Thaut et al. 1993, 1996). Starting from the studies by Thaut and colleagues, a large body of research investigated the efficacy—in the rehabilitation context—of different types of auditory interventions, including sonification. Various sonification approaches have been used; for instance, it has been shown that sonification in combination with action observation has beneficial effects on Parkinson’s symptoms (Mezzarobba et al. 2018) and that real-time sonification can enhance knee re-positioning accuracy (Ghai et al. 2018).

## 3. The Ecological Sound Approach

As effectively summarized by Schaffert et al. (2019), ecological (natural) movement sounds “carry rich auditory information that has direct physical correspondence to their referent event(s), providing crucial information that may be used to inform or enhance task-intrinsic feedback” (p. 2). One technique drawing on such a richness of information is auditory modeling, which consists of the use of auditory recordings of the sounds produced during the execution of a complex, rhythmic movement as models; these models contain and convey information regarding the rhythm, duration and intensity of the motor act in an ecological format (Agostini et al. 2004). The idea behind this technique is that athletes can benefit from information conveyed through ecological sounds and reproduce movements with similar features. Before describing the procedure in more detail, as well as mentioning some applications of this technique to sport and rehabilitation, we briefly define perceptual modeling in general.

### 3.1. Perceptual Modeling in Motor Activities

Perceptual modeling is a widely used technique that relies on learning by imitation. Its psychological underpinnings come from Bandura’s (1969, 1971) Social Learning Theory on the one hand, and from Rizzolatti and colleagues’ discovery of the functioning of mirror neurons on the other (Fadiga et al. 1995; Kohler et al. 2002). It is a way of accumulating experience before experiencing it directly; it suggests new behavior and projects the performers into new situations, helping them to build progressively more precise and detailed mental images of the motor scheme to be learnt.

Modeling is a “family” of many kinds of intervention strategies; consequently, depending on the kind of protocol chosen, the methods can be quite different, and the target movements to be trained can have different levels of complexity. We may generally categorize these methods into two main branches based on the perceptual channel being used, i.e., visual modeling techniques and auditory modelling techniques.

Although visual perception is usually predominant (e.g., Posner et al. 1976) in the organization and control of movement, in sports and/or motor activities characterized by a cyclic repetition of the same movements, such as swimming, walking, running, etc., visual information is of little help with respect to auditory information. In fact, it has been shown that auditory models, i.e., sequences of sounds that reproduce the timing of a given movement, are more effective than visual models in promoting the identification, discrimination, memorization and reproduction of precisely timed movements (e.g., Doody et al. 1985; Glenberg and Jona 1991; Grondin and McAuley 2009; Lai et al. 2000). Therefore, when dealing with complex movements (for instance, athletic and technical gestures in sport), it is worth concentrating on auditory modeling.

### 3.2. Auditory Modeling

#### 3.2.1. The Procedure

As mentioned above, auditory modeling consists of the use as models of recordings of ecological sounds produced during the execution of a movement. Specifically, the procedure for this technique comprises the following steps: (1) recording the ecological sounds produced during a series of repetitions of the same movement executed by a performer (e.g., the sound of rotations in a hammer throw); (2) selecting the sound track associated with the best performance, both in terms of objective performance outcome and the subjective evaluation of gesture execution; (3) administering the selected sound track to the performer, asking her/him to mentally represent the execution of the movement while listening to the auditory model; and (4) performing a new series of repetitions (less commonly, sound can be administered during movement execution).

Since ecological auditory rhythm is a natural result of human movement, it can be used as a guide for human action. In order to develop an effective training strategy, we believe that it is necessary to start from the performer’s phenomenal experience, i.e., the perceptual experience deriving from the sound produced by her/his own movement. In fact, each athlete/patient moves in a different way, and therefore produces a different pattern of sounds. Auditory modeling is a specific way of translating this personal phenomenal experience into a cognitive strategy aimed at improving/standardizing the performance.

#### 3.2.2. Applications to Sport

To the best of our knowledge, the first example of the effectiveness of auditory modeling in sport was provided by Agostini et al. (2004), with a study on the hammer throw. On the first day, the participants—expert throwers—were asked to perform two sets of ten throws each to rule out the presence of possible confounding variables as a negative effect of fatigue or a positive effect of practice on performance. On the second day, the participants performed another two series of ten throws each. During the first series, a microphone was placed near the head of the hammer to record the sound produced during the rotation movement. This series also served as a baseline. At the end of this first series of throws, each athlete listened individually to the ten acoustic tracks s/he produced and was then asked to choose the track that s/he thought was associated with her/his best throw. All the athletes correctly identified the sound track produced by their best personal throw out of the ten they listened to. The second series was the experimental phase, in which the sound associated with each athlete’s best throw was used as a model and administered five times before each of the ten throws of the second series. The results highlighted a twofold improvement in performance: compared to the baseline series throws, those of the experimental series were on average significantly longer, and their variability was significantly lower.

Another sport in which auditory modeling has proven to be effective is hurdling. After an initial study based on real-time ecological auditory feedback (Kennel et al. 2015), Pizzera et al. (2017) examined the short- and long-term effects of a training protocol based on offline auditory feedback. In particular, in addition to a group with “standard” models similar to those of Agostini et al. (2004), there were two more groups, i.e., one with models played back with an increase in the tempo and one with models played back with a decrease in the tempo. In the short term, performance—in terms of both running time and movement technique—improved for all three groups; however, in the long term, only the groups with faster and slower tempos showed further improvements, while the group with normal tempos showed a decline in performance.

#### 3.2.3. Applications to Motor Rehabilitation

Like sonification, the use of ecological sounds also turned out to be effective not only in sports but in motor rehabilitation, too. For example, Murgia et al. (2018) conducted a randomized controlled trial with Parkinson’s disease patients, assigning some of them to “classic”, metronome-based RAS training and some others to training based on ecological sounds consisting of recordings of footsteps. After intensive training lasting five weeks, the patients of both groups showed significant improvements in the majority of the clinical and biomechanical parameters considered; moreover, these improvements were maintained at a follow-up three months later. Interestingly, even if by comparing the two groups no differences emerged between them, exploratory analyses conducted considering the two groups separately highlighted statistically significant improvements in cadence (from pre-test to post-test) and gait speed (from pre-test to post-test and in the follow-up) only for the group training with footsteps sounds, while no significant improvements in these variables were observed in the metronome group. Although this evidence cannot be considered conclusive, these outcomes are consistent with further evidence suggesting the potentially higher efficacy of ecological sound-based approaches (e.g., Rodger et al. 2014; Young et al. 2014, 2016). Altogether, these studies indicate the potential of ecological sounds for motor rehabilitation. However, we acknowledge that this topic is still under-researched; thus, further investigation is needed to better explore the practical applications of this approach.

## 4. Comparing the Approaches: Current Knowledge and Future Perspectives 

In the two previous sections, we saw that an appropriate use of sounds deriving from one’s own movement can promote significant improvements in movement execution itself, both for sport performances and motor rehabilitation. In particular, such an appropriate use can follow two main approaches, i.e., the sonification one or the ecological sound one. The former is based on the conversion of physiological and/or physical movement data into sound(s) and has proven to be effective in sports like swimming, cycling and rowing, as well as in different rehabilitation processes. The latter is based on the use of auditory recordings of movement sounds as models and has proven to be effective in sports like hammer throw and hurdling, as well as in gait rehabilitation.

Between the sounds used in the sonification approach and those used in the ecological sound approach, there is a fundamental difference; the former are artificial sounds while the latter are ecological sounds. It follows that, whereas with the sonification technique the same physical/kinematic parameters can be converted into different sounds as long as the parameters one decides to represent remain unchanged, with the ecological sound approach all parameters potentially relevant to performance are already present.

Both neurophysiological evidence and perceptual—motor theories seem to support the effectiveness of interventions based on the administration of auditory information. As regards the neurophysiological evidence, it is well known that neurons with mirror properties are associated with imitation; according to the perceptual—motor theories, the perceptual and motor systems share a common representational organization, and they continuously influence each other (for further theoretical implications, see Agostini et al. 2020; Sors et al. 2015).

The effectiveness of auditory information interventions encourages us to further explore their potential; in particular, three relevant future directions can be identified. One is mainly related to sports: it would be interesting to compare the two approaches between novices and experts, to see whether or not one approach is more effective than the other based on the level of experience/expertise in a specific discipline; this would allow us to adopt the most appropriate approach for training purposes. The other two future directions are relevant both for sport and rehabilitation: on the one hand, it would be useful to understand to what extent non-cyclic movements could also benefit from these approaches; on the other hand, the number and duration of training sessions needed to promote consolidated enhancements and whether or not beneficial effects last on a long-term basis should be further investigated. As research will shed light on these important aspects, the use of these approaches in applied practice will become more and more common.

As for applications, it is evident that the two approaches have different pros and cons. Sonification can also be used to provide feedback on movements that naturally do not produce any sound, and it has a certain degree of flexibility in terms of both the parameters of movement to be sonified and the auditory dimension used as feedback. However, sonification requires expensive equipment and technical expertise, which make its use quite difficult in applied sport/rehabilitation contexts. Furthermore, information conveyed through sonification may be perceived as “artificial”, and its use in applied contexts may require some practice. Conversely, ecological sound interventions are mainly limited to those movements that produce some (at least slightly audible) sound; the stimuli can be adjusted in the laboratory, but the flexibility of these interventions is reduced compared to those based on sonification. However, the technical skills and equipment needed to implement these interventions make them more usable and adaptable to different applied contexts. Moreover, the ecological stimuli—being more “natural” than sonification by definition—should more easily elicit a mental representation of action, possibly making their use in applied contexts more immediate.

One could argue as to whether or not athletes can be trained using sound in order to gain an actual advantage in real sport competitions. Perhaps the first thing that should be clarified is whether or not athletes already use auditory information (e.g., Cañal-Bruland et al. 2022). If so, this type of training would aim to enhance existing cognitive strategies rather than induce the development of new ones. The use of auditory information in real-life sports situations was first investigated by Takeuchi (1993) in his seminal study, in which he found a decline in performance when participants played tennis without auditory information. Although the small number of participants limits the generalizability of these results, they suggest that athletes may use auditory information during performance. In this regard, Schaffert et al. (2020) provided more recent and compelling evidence in rowing, showing that objectively measured movement precision decreased, and subjectively measured cognitive costs increased, when auditory information was not present during performance. Therefore, it is likely that athletes—more or less implicitly—use ecological sounds (when available) and integrate them with other sources of information to mentally represent the surrounding environment and interact with it.

So, what happens when auditory information is removed during sport performance? Although Gestalt psychologists have mainly dealt with visual perception (Koffka 1935; Wertheimer 1923), some of them also extended Gestalt principles to multisensory integration (see, for example, Werner 1934). They would probably say that the “whole” experience (i.e., the multisensory integration of information) is somehow disrupted when a piece of information is removed, even if its contribution to the “whole” appears limited. In conclusion, as with most topics in science, we have no definitive answer to the original questions posed in this article. However, the evidence accumulated over the past 18 years regarding the relevance of ecological sounds in sport leads us to lean toward a positive answer.

## Data Availability

Not applicable.
